# Controlling the Electronic Properties of a Nanoporous Carbon Surface by Modifying the Pores with Alkali Metal Atoms

**DOI:** 10.3390/ma13030610

**Published:** 2020-01-30

**Authors:** Michael M. Slepchenkov, Igor S. Nefedov, Olga E. Glukhova

**Affiliations:** 1 Department of Physics, Saratov State University, Astrakhanskaya street 83, 410012 Saratov, Russia; slepchenkovm@mail.ru; 2 School of Electrical Engineering, Aalto University, P.O. Box 13000, 00076 Aalto, Finland; igor.nefedov@aalto.fi; 3 Faculty of Science, People’s Friendship University of Russia (RUDN University) 6 Miklukho-Maklaya St, 117198 Moscow, Russia; 4 Laboratory of Biomedical Nanotechnology, I.M. Sechenov First Moscow State Medical University, Bolshaya Pirogovskaya street 2-4, 119991 Moscow, Russia

**Keywords:** nanoporous carbon surface, electronic structure, emission properties, work function, potassium atoms, charge transfer

## Abstract

We investigate a process of controlling the electronic properties of a surface of nanoporous carbon glass-like thin films when the surface pores are filled with potassium atoms. The presence of impurities on the surface in the form of chemically adsorbed hydrogen and oxygen atoms, and also in the form of hydroxyl (OH) groups, is taken into account. It is found that even in the presence of impurities, the work function of a carbon nanoporous glass-like film can be reduced by several tenths of an electron volt when the nanopores are filled with potassium atoms. At the same time, almost all potassium atoms are ionized, losing one electron, which passes to the carbon framework of the film. This is due to the nanosizes of the pores in which the electron clouds of the potassium atom interact maximally with the electrons of the carbon framework. As a result, this leads to an improvement in the electrical conductivity and an increase in the electron density at the Fermi level. Thus, we conclude that an increase in the number of nanosized pores on the film surface makes it possible to effectively modify it, providing an effective control of the electronic structure and emission properties.

## 1. Introduction

It is well known that carbon nanoporous glass-like materials are actively used in modern emission electronics [1,2,3,4,5,6]. In particular, they are very promising for the manufacture of field emission cathodes based on them. Such cathodes exhibit promising field emission parameters [7,8,9]. The edges of pores in these carbon glass-like materials are sharp blade structures that make them emission centers with a field enhancement factor, β. As already known, the emission tips of matrix cathodes with a high emission current density are traditionally produced on the basis of this mechanically strong material. Carbon glass-like materials have high strength, high heat resistance, abrasion resistance, and chemical inertness, as well as isotropic electrical conductivity [10,11]. It should also be noted that modern technology allows us to control the size of nanopores. For example, by forming nanoporous carbon on a conductive substrate, the nanopore size can be varied from 0.6 to 4.0 nm [12,13]. The samples of such nanoporous carbon were produced as a result of thermochemical treatment of different carbides with chlorine at different temperatures. It should also be noted that interest in the glass-like carbon nanomaterial increases in connection with the increasing possibilities of modifying its pores with atoms of various chemical elements [14,15,16]. With the increased capabilities of computer modeling, it has become possible to study all the features of carbon surface modification at atomic level [17]. The atomic structure of the glass-like carbon nanomaterial was reproduced due to modern microscopy. The glass-like carbon can be divided into two types: anisotropic material of multi-layer graphene flakes forming a likeness of layers (graphitized glass-like carbon) and randomly arranged graphite flakes (non-graphitized glass-like carbon) [18,19]. Non-graphitized glass-like carbon, in turn, can be divided into two classes: the first class has mainly randomly distributed curved layers of graphene fragments [20], the second class has self-organizing nanometer fullerene-like spheroids and 3D disordered multilayer graphene flakes [21]. In some recent papers, the carbon material between few-layer graphene and fullerenes are also called porous glass-like carbon [22].

**Figure 1:**
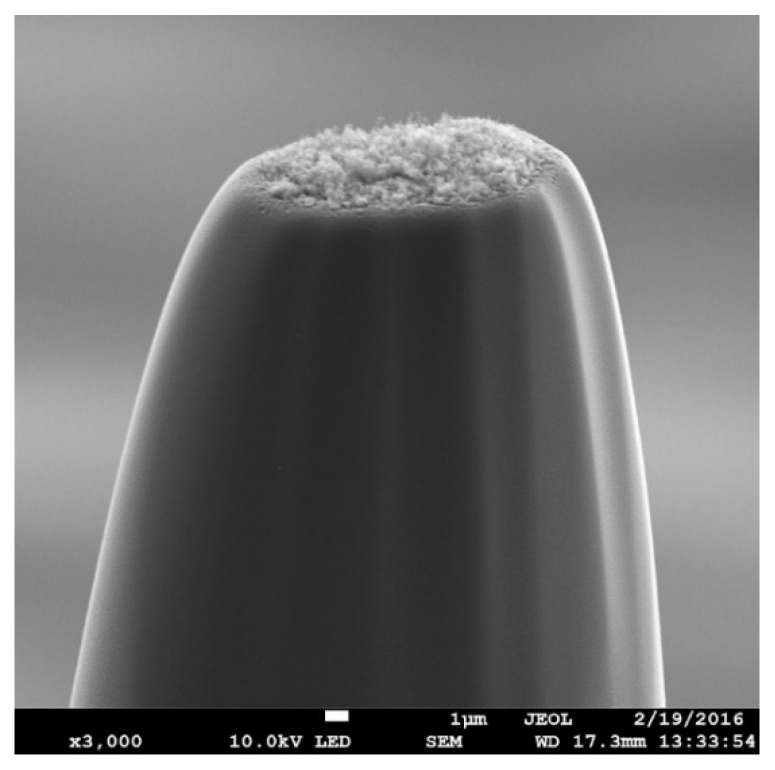
SEM image of the emitting tip surface of the porous glass-like nanomaterial.

At the same time, the question of the effect of the emitting tip surface on the emission properties remains topical. At present, it is possible to modify and nanostructure the surface of porous carbon glass-like materials [2], which makes it possible to sharply increase the field enhancement factor β by 4–5, because a thin surface layer of the emitting material plays a decisive role in autocathodes. This layer may be compared with a film of a thickness of several tens of nanometers, as shown by Gay et al. [2]. A typical scanning electron microscope (SEM) image of the emitting surface of a nanoporous carbon glass-like emitter is shown in Figure 1. This image was taken using a JEOL JEM-2100 Plus transmission electron microscope at an accelerating voltage of 10 kV. The diameter of the tip at the base is 32 microns; the diameter of the apex is 16 microns. As can be seen in the figure, the working surface of the emitting tip is a rough structure which ensures a high density of field emission current. The technology for producing such films using laser radiation is described in detail by Bessonov et al. [23]. 

However, the complexity of the surface modification problem is that the surface always contains at least a small number of impurity atoms [24]. First of all, these are atoms of oxygen, hydrogen, and OH groups. Even despite the technical vacuum conditions (10^–6^–10^–7^ Torr) under which autocathodes work in most cases, a certain proportion of adsorbates remain. The presence of impurities inevitably leads to a deterioration of the emission properties [25,26]. However, there is a method for an efficient surface modification, for example, by doping with alkali metal atoms, which contribute to a decrease in the work function [27,28]. Thus, the emitter surface plays the most important role in all emission processes [29,30]. Depending on the type of impurities adsorbed on the emitter surface, as well as the surface topology, the emission properties of the material may deteriorate or improve [31,32,33,34,35,36]. Moreover, the impurities and topology determine the energy of the surface and the electron charge density distribution. The combination of these factors determines the work function and the electrical conductivity of the emitting surface. 

This work is devoted to the in silico study of the patterns of change in the electronic properties of thin films of carbon nanoporous glass-like nanomaterial upon oxidation and modification by hydroxyl groups and potassium atoms, from the standpoint of the effect on the emission properties and the expansion of the applications of this nanomaterial in electronics. As the carbon nanoporous film, we considered a film with a thickness of 4–5 nm, whose surface had a nanopore system formed by the fragments of single- and few-layer graphene flakes and fullerenes. 

## 2. Atomistic Model of the Nanoporous Carbon Surface

**Figure 2:**
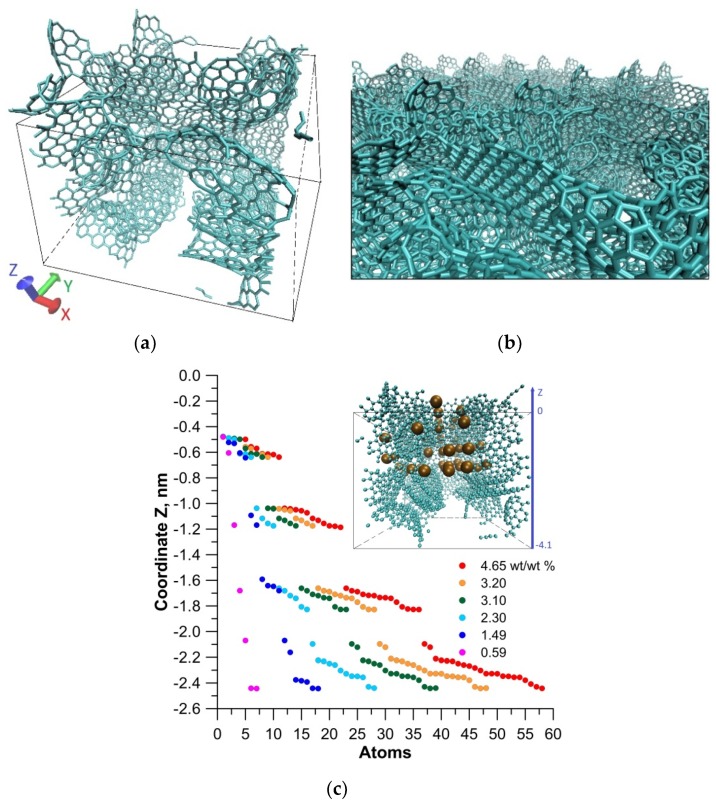
A surface of porous glass-like nanomaterial: (**a**) a super-cell of the film, which is the glass-like nanomaterial surface; (**b**) a topology of the atomic model surface; (**c**) a distribution of potassium atoms (balls of ocher color) by nanopores at different concentrations (mass fraction).

A super-cell of porous glass-like carbon nanomaterial film was constructed for this investigation. Earlier, we had created an atomistic model of a porous glass-like carbon nanomaterial [37], which was a combination of interacting graphene flakes and fullerene fragments (see Figure 2a). The atomic network of the super-cell was obtained as a result of the optimization of the atomic cell by the self-consistent charge density functional tight-binding (SCC-DFTB) method [38]. The calculations were performed using the DFTB+ package [39]. This program uses one of the best parameterizations of the SCC-DFTB method. This parameterization is very popular in academia. According to Google, it is mentioned in more than 20,000 scientific publications as a calculation method. A comparative analysis of the results of using density functional theory (DFT) and DFTB, using the example of defective graphene, showed that the DFTB method reproduced both the structural and energy parameters of nanostructures with high quantitative accuracy [40]. The error of the SCC-DFTB method depended on the class of problems being solved. In particular, the error in calculating the atomic structure and electronic parameters (energy gap of the band structure, Fermi level, and distribution of the electron charge over atoms) was several percent. A super-cell of 2D porous carbon material was a thin layer of glass-like nanomaterial, the surface of which corresponded to the equilibrium state. The approach of super-cell formation had previously been used by the authors to construct a bulk sample of a porous glass-like material. As a result, the structural density of the created film model was 1.21 g/cm^3^ with the dimensions of the super-cell 4.2 nm × 4.2 nm × 4.1 nm. As the film was a two-dimensional structure, the super-cell of the film was only translated in the X and Y directions, as shown in Figure 2a. The number of atoms in the super-cell was 3874 (not taking into account the impurity). The pore size in our model was 0.25–1 nm, which corresponded completely to the glass-like porous nanomaterial [19,21]. The fraction ratio of non-hexagonal elements was 9.2%, which also fully corresponded to the structure of the glass-like porous nanomaterial [21], which was characterized by 10%–15% of non-hexagonal elements. The super-cell translated in the X and Y directions reproduced the surface of the emitting tip with some approximation. In the Z direction, the structure had freely oriented fullerene fragments and graphene flakes. The atomic networks of these fragments were obtained as a result of optimization by the SCC-DFTB method. The surface of the porous film obtained on the basis of the created super-cell is shown in Figure 2b. If we compare the obtained surface with the SEM image shown in Figure 1, one can see the similarity of topology, which is represented by a large number of individual fragments of nanostructures. The developed surface of the atomistic model of a nanoporous carbon material had numerous fragments of graphene and fullerene flakes. Nanopores were located on the surface, no deeper than 2.6 nm. 

The modification of the surface with potassium atoms was carried out as a result of bombarding the surface of the glass-like carbon with potassium atoms using the molecular dynamics (MD) SCC-DFTB method under normal external conditions [41]. The calculations were performed using open-source Kvazar [42]. The initial conditions were taken from experimental data [43], in which the beam energy was from 50 to 150 keV at a beam density of 10^15^–10^17^ atom/cm^2^. We considered the case when the beam density was 10^16^ atom/cm^2^, and the energy was equal to 100 keV. The velocity of the atoms was 0.70 m/s under such conditions. The main regularities in filling the pores and reaching the maximum potassium concentration were found as a result of a series of 20 numerical experiments. This process was accompanied by temperature fluctuations within 300–800 K. It was established that the maximum possible potassium concentration by mass was 4.65% for this structure of glass-like carbon surface. Figure 2c shows the course of filling the nanopores with potassium atoms. The ordinate represents the depth at which the nanopores were located (negative direction of the Z axis) and the abscissa—the number of potassium atoms filling these nanopores. One can see a clear regularity in the filling of the nanocavities of the glass-like carbon surface. Even at a low concentration of 0.59%, two potassium atoms reached a depth of 2.5 nm with respect to the surface layer. During the process of bombarding the surface of glass-like carbon, all the nanocavities were uniformly filled with potassium atoms. This is well demonstrated by the inset of Figure 2c, which corresponded to the maximum concentration, where the potassium atoms uniformly filled the surface, deep, and middle cavities (potassium atoms are marked with ocher-colored balls). Thus, all pores were filled at a depth of 2.5–2.6 nm.

## 3. Results and Discussion 

As already known, the working nanostructured surface of the emitting tip is not absolutely clean. It always contains adsorbed oxygen atoms, hydrogen, OH groups, or a combination of these. These impurity atoms/atomic groups always increase the work function of the material, especially oxygen. In connection with this, we conducted an in silico investigation of various combinations of impurity elements with different concentrations.

**Figure 3:**
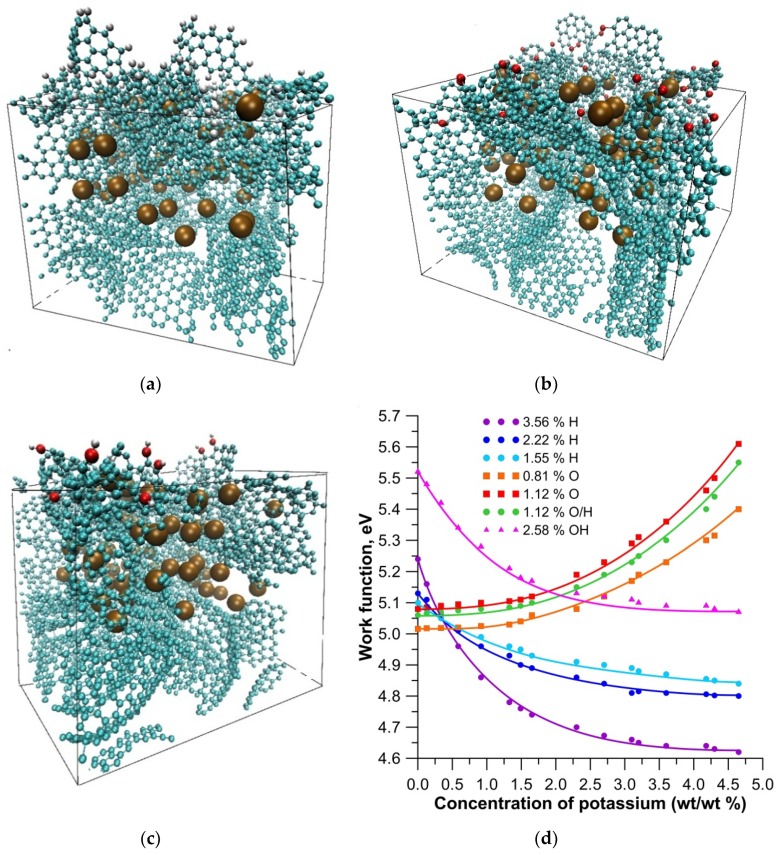
The atomic structure and the work function of a nanocarbon porous film with different types of impurity atoms: (**a**) modification by hydrogen atoms (3.56%); (**b**) modification by oxygen atoms (0.81%); (**c**) modification by OH groups (2.58%); (**d**) a graph of the change in the work function with an increase in the mass fraction of potassium at different concentrations of impurity atoms.

One series of numerical experiments was carried out by filling the nanopores with potassium atoms during the hydrogenation of fragmentary surface elements. Figure 3a shows a super-cell of a nanocarbon film whose surface contained 3.56% atomic hydrogen (hydrogen atoms are shown in white and gray color). Hydrogen atoms were located on the edges of the atomic network fragments, because these atoms of the carbon framework were most chemically active. Balls of ocher color represent potassium atoms. It should be noted that the impurity atoms of hydrogen did not change the process of filling the nanopores with alkali metal atoms. The directions of the X, Y, and Z axes remained the same, as shown in Figure 2c. We constructed super-cells and calculated the band structures and densities of states (DOS) with determination of the Fermi level for various values of the atomic hydrogen concentration. Numerical simulations of the process of filling the nanopores with potassium atoms were performed for each case until the maximum concentration was achieved. Noticeable changes in the energy of the film and, in particular, in the position of the Fermi level, which determines the work function, were detected during the filling of the nanopores. We found that the work function of the film decreased as the nanopore was filled with potassium atoms regardless of the amount of adsorbed atomic oxygen. When the initial value of the work function was 5.1–5.3 eV, then it amounted to 4.6 eV with the maximum filling of the nanopores. This result was extremely important for the process of electron emission by the application of an electric field. It is characteristic that a lower value of the work function was achieved with maximum hydrogenation of the film surface in this case. It should also be noted that the work function of the film with the surface adsorbing the impurity atoms (4.96 eV) was always greater than the work function of the pure carbon framework. We noted that a decrease in the work function during hydrogenation was provided by an increase in the potassium concentration at a given fixed fraction of hydrogen atoms on the surface. We can say that the hydrogenation did not prevent a reduction in the work function during the filling of pores by potassium atoms. 

Another series of experiments was devoted to the study of the band structure, the position of the Fermi level, the oxidation of the surface, the adsorption of OH groups, and also the adsorption of oxygen atoms, hydrogen, and OH groups simultaneously. Figure 3b shows the super-cell at the oxidation (oxygen atoms are shown in red) of the surface fragments of the atomic cell and at the maximum filling of the nanopores with potassium. The concentration of oxygen atoms was 0.81%. Figure 3c shows the super-cell of the film upon adsorption of OH groups on its surface (0.58%). The band structures and Fermi levels were calculated for all cases of different concentrations of impurity atoms. The analysis showed that the type of impurity directly determined the nature of the change in the energy structure and the position of the Fermi level. Figure 3d shows a graph of the work function as a function of the mass fraction of potassium for various variants of impurity atoms and their concentration. Characteristic groups of curves were distinguished. Firstly, all cases with hydrogenation were characterized by a decrease in the work function when filling the nanopores with potassium. 

As can be seen in Figure 3d, with an increase in the concentration of hydrogen atoms on the surface, the work function decreased against the background of increasing potassium mass fraction. Secondly, oxidation impaired the emission properties of the surface regardless of the concentration of oxygen atoms. The work function only increased, in particular by 0.5–0.6 eV, regardless of the number of oxygen atoms and the degree of presence of potassium atoms. Other variants of impurity atoms showed a noticeable deterioration of the emission properties, regardless of the concentration. The results of the calculations showed that the presence of OH groups increased the work function by 0.5 eV, in comparison with hydrogen. However, the modification of pores with potassium made it possible to compensate increasing work function, as can be seen in Figure 3d (curve with triangles). It should also be noted that the presence of both O atoms and H atoms on the surface simultaneously, as with oxidation, only contributed to an increase in the work function.

In order to find the physical phenomena that occurred during such changes in the energy structure of the film and in the displacement of the Fermi level, we investigated the charge transfer between atoms of potassium and the carbon framework with adsorbed atoms/atomic groups. Table 1 shows the calculated values of Mulliken charges—charges localized on the carbon framework, on potassium (K) atoms, and on other atoms of the surface. The same cases of impurity atoms on the surface are given as in Figure 3d. Potassium atoms always gave up their charge. The dependence of the amount of overflowing charge from potassium atoms on the carbon framework was non-linear. An analysis of the pattern of the electron density distribution showed that the potassium atoms lost almost an entire electron (0.8–0.9 e) directly near the framework and that the atoms inside the group of potassium atoms practically did not lose anything (they had a charge of 0.26 e). Therefore, the ratio of the charge to the number of potassium atoms in the cell varied from 0.86 to 0.73 e/atom.

**Figure 4:**
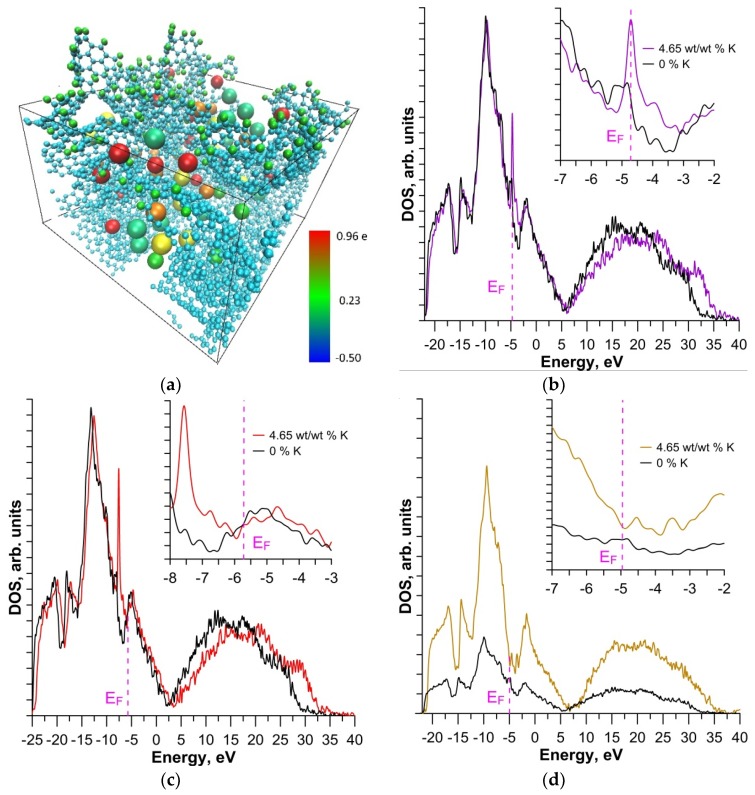
Distribution of the electron charge: (**a**) on a surface modified by hydrogen atoms with a concentration of 3.56%, with a maximum mass fraction of potassium; (**b**) Density of states (DOS) of hydrogenated (3.56%) surface without potassium (gray curve) and with potassium (lilac); (**c**) DOS of oxidized (1.12%) surface without potassium (gray curve) and with potassium (red); (**d**) DOS of a clean surface without potassium (gray curve) and with potassium (ocher color).

Figure 4a shows a super-cell with impurity hydrogen atoms (3.56% H) and a maximum mass fraction of 4.65%. The charge is displayed in color—the potassium atoms change color from red (charge +0.96 e) to yellow green (+0.50 e) and green (+0.26 e). The carbon framework had a practically uniformly distributed charge −0.1 e to −0.5 e, therefore it is displayed in blue. Hydrogen atoms, like potassium, gave up a partial charge, so they are shown in green. Their charge varied from +0.1 to +0.23 e. Thus, analysis of the electron charge density distribution data (data in Table 1) and comparison with the graphs in Figure 3d shows that the minimum work function was observed for the case of the maximum transfer of electron charge to the carbon framework. This was due to an increase in DOS at the Fermi level. Figure 4b shows DOS for the case of a maximum displacement of the Fermi level upward and a corresponding decrease in the work function (lilac curve). It can be clearly seen that in this case the DOS peak fell on the Fermi level, which improved electrical conductivity and reduced the work function. The gray color represents DOS surfaces with 3.56% H, but without potassium atoms. When oxidizing the surface, even pouring potassium into the pores did not improve the emission properties. Figure 4c shows DOS for the oxidized surface with a concentration of 1.12% O (gray curve) and for the same surface at the maximum mass fraction of potassium (red curve). It can be seen that at the Fermi level the DOS of the surfaces with potassium were smaller in comparison to the case without potassium. For comparison, we calculated the DOS of a clean surface (without impurity atoms) and a surface with the maximum mass fraction of potassium. These calculation results are presented in Figure 4d. The DOS increased at the Fermi level. 

**Figure 5:**
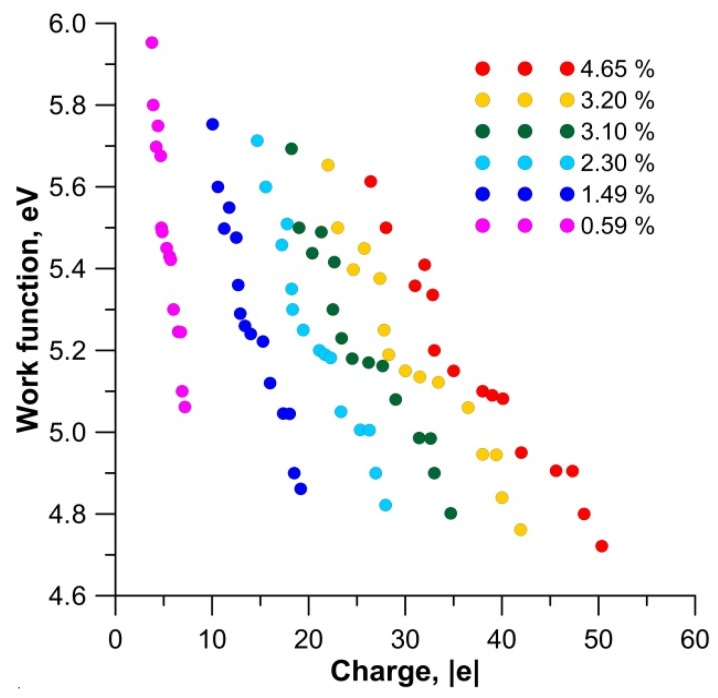
The change in the work function of the film with an increase in charge on the carbon framework for various cases of filling the nanopores with potassium.

Calculations of the charge of the carbon framework in all considered cases of surface modification showed that there was a clear regularity. As the electron charge flowed over the carbon framework, the work function decreased. Figure 5 shows the graphs of the change in the work function of the film with an increase in charge on the carbon framework for various cases of filling the nanopores with potassium. 

## 4. Conclusions

Regularities in the electronic structure and charge transfer in the nanoporous surface of glass-like thin films when the surface pores were filled with potassium atoms were investigated using MD DFTB calculations. We constructed an atomistic model of the porous carbon film surface, completely reproducing the features of the glass-like porous nanomaterial structure. It was found that the maximum possible concentration of potassium by weight was 4.65% for the examined glass-like carbon surface. In this case, the potassium atoms uniformly filled the surface, deep, and middle cavities at a depth of 2.5–2.6 nm. The presence of impurities in the form of chemically adsorbed hydrogen atoms, oxygen, and OH groups was taken into account during the calculation of the electronic structure of the glass-like carbon surface doped with potassium. The calculation results showed that the type of impurity determines directly the nature of the change in the band structure and the position of the Fermi level of the glass-like carbon surface doped with potassium. Hydrogen saturation reduces the work function, oxidation increases the work function by 0.5–0.6 eV regardless of the oxygen concentration, and, finally, the presence of OH groups increases the work function by 0.5 eV, compared with hydrogen. When the surface nanopores filled with potassium atoms, all the potassium atoms were ionized, losing one electron, which transferred over to the carbon framework of the film. It was found that changing the mass fraction of potassium atoms on the surface can reduce the work function even in the presence of impurity atoms, which impair the emission properties.

## Figures and Tables

**Table 1:** Charge distribution at the maximum mass fraction of potassium (4.65%).

N/N _atoms_, %	Charge, |e|				
	K	H	O	OH	Carbon
3.56% H	+39.95	+10.39	-	-	−50.34
2.22% H	+40.20	+7.10	-	-	−47.30
1.55% H	+40.46	+5.14	-	-	−45.60
0.81% O	+41.28	-	−10.38	-	−30.90
1.12% O	+41.45	-	−15.04	-	−26.41
2.58% OH	+40.10	-	-	−1.20	−38.90
1.12% O, 1.12% H	+40.75	+1.60	−8.42	-	−33.93
